# Immunological Crossroads: Optimizing Antirejection Regimens to Sustain Antitumor Immunity in Liver Transplant Recipients with Hepatocellular Carcinoma

**DOI:** 10.3390/cancers17233871

**Published:** 2025-12-02

**Authors:** Chao Zhang, Xin Yuan, Kunlin Xie

**Affiliations:** 1 Department of General Surgery, West China Hospital, Sichuan University, Chengdu 610041, China; 19508387607@163.com (C.Z.);; 2 Liver Transplant Center, Transplant Center, West China Hospital, Sichuan University, Chengdu 610041, China

**Keywords:** liver transplantation, hepatocellular carcinoma, immunosuppression therapy, immunotherapy, tumor microenvironment

## Abstract

Liver transplantation (LT) is a curative treatment for hepatocellular carcinoma (HCC). However, lifelong immunosuppressive therapy required to prevent graft rejection inevitably compromises antitumor immunity, thereby increasing the risk of HCC recurrence and metastasis, particularly common in the lungs. This review delves into the complex dynamic equilibrium between immune cell subsets mediating rejection and antitumor immunity, systematically analyzes the impact of current immunosuppressive regimens on this balance, and highlights emerging strategies aimed at minimizing rejection while preserving or enhancing antitumor efficacy. These strategies include immunosuppressive regimen optimization, such as mTOR inhibitor application and calcineurin inhibitor (CNI) minimization, novel immunotherapies, including immune checkpoint inhibitors (ICIs) and adoptive cell therapy (ACT), and immune tolerance induction. This review also summarizes advances in biomarker research guiding immunosuppressant withdrawal, aiming to provide a theoretical basis and clinical insights for personalized immunotherapy strategies and comprehensive tumor management in LT recipients with HCC.

## Simple Summary

Liver transplantation offers a curative option for hepatocellular carcinoma (HCC), but its long-term success is challenged by a fundamental therapeutic dilemma: the essential immunosuppressive drugs that prevent graft rejection simultaneously impair the body’s natural defenses against cancer recurrence. This review examines the intricate balance between rejection control and antitumor immunity, evaluating how conventional and emerging strategies—from optimized immunosuppression regimens to mTOR inhibitors and adoptive cell therapy—can be tailored to protect the graft while minimizing oncological risk. By synthesizing recent advances in this evolving field, we aim to inform more precise and individualized approaches to post-transplant care, ultimately improving survival and quality of life for HCC transplant recipients.

## 1. Introduction

Hepatocellular carcinoma (HCC) is one of the most significant global health challenges. According to the latest cancer epidemiological data, liver cancer ranks sixth in incidence and third in mortality worldwide [1]. In China, the disease burden of HCC is particularly heavy, being a leading cause of cancer-related death [2]. Liver transplantation (LT) provides the best curative opportunity for eligible HCC patients by simultaneously removing the tumor lesions and the underlying cirrhotic liver, achieving 5-year survival rates exceeding 70% [3]. However, post-transplant HCC recurrence remains a major obstacle to long-term survival, with a 5-year recurrence rate of up to 4.3% even in patients strictly meeting the Milan criteria [4]; once recurrence occurs, the median survival time drops sharply to 10.6–12.2 months [5].

Metastasis is a key factor for poor prognosis in HCC, with the lungs being the most common site of extrahepatic metastasis, accounting for approximately 60% of all metastatic cases [6]. The prognosis for HCC patients with lung metastasis is extremely poor; studies show untreated patients have 1-year, 3-year, and 5-year overall survival rates of only 12.8%, 4.0%, and 1.6%, respectively [7]; another cohort study reported a median survival time from diagnosis of lung metastasis to death as short as 3.3 months [8]. These data underscore the urgency of clinical intervention and the severe challenges in treating HCC metastasis.

To prevent graft rejection, LT recipients require lifelong immunosuppressive therapy [9]. Nevertheless, such non-specific immunosuppressive regimens, while protecting the graft, also broadly weaken immune surveillance, increase infection risk, promote de novo malignancies, and may elevate the risk of tumor recurrence [9,10]. This constitutes the core clinical dilemma in managing these patients: how to effectively prevent allograft rejection while maximally preserving or even enhancing the host’s immune capability against tumors [10].

This review aims to deeply dissect the cellular and molecular basis of this immunological balance, systematically evaluate the impact of current immunosuppressive strategies on HCC recurrence risk, and explore emerging therapeutic strategies designed to optimize this balance. The article will focus on the dual roles of immune cells in rejection and antitumor immunity, analyze the unique characteristics of the liver’s immune microenvironment, evaluate the pros and cons of existing immunosuppressive protocols, and comprehensively discuss various strategies, including immunosuppressive regimen optimization, application of novel immunotherapies, and immune tolerance induction, to provide an evidence-based foundation and new ideas for clinical practice.

For the purpose of this review, a systematic literature search was conducted utilizing the PubMed, Embase, and Web of Science databases. To ensure the inclusion of the most recent and emerging data, searches were also performed in major international conference proceeding libraries, covering the period from their inception to 28 September 2025. The search strategy was built upon core keyword combinations, including “liver transplantation,” “hepatocellular carcinoma,” “immunosuppression,” “immunotherapy,” “immune checkpoint inhibitors,” “adoptive cell therapy,” “rejection,” “tumor microenvironment,” and related terms.

## 2. Bidirectional Regulatory Roles of Immune Cells in Graft Rejection and Antitumor Surveillance

The maintenance of immune homeostasis after liver transplantation relies on the precise regulation of immune cell function. However, most immune cells not only mediate allograft rejection but also undertake antitumor immune surveillance functions. This functional overlap constitutes the core contradiction in clinical immune management: suppressing rejection may weaken antitumor defense, while preserving antitumor immunity may increase rejection risk. A deep understanding of the dual roles of various immune cells at this immunological crossroads is the theoretical foundation for developing precise therapeutic strategies.

### 2.1. T Lymphocytes: Balancing Effector and Regulatory Functions

T cells play a central role in both transplant rejection and antitumor immunity. In rejection, CD4^+^ T cells recognize allogeneic MHC class II molecules on the surface of donor antigen-presenting cells (APCs), and differentiate into helper T cells, such as Th1 and Th17, driving inflammatory responses and activating CD8^+^ cytotoxic T cells (CTLs) [10,11,12]. Activated CTLs kill graft cells via the perforin-granzyme and Fas/FasL pathways [11]. Recent studies indicate that some effector CD8^+^ T cells differentiate into tissue-resident memory T cells (TRMs) within the graft, surviving long-term and continuously expressing effector molecules and immune checkpoint proteins, becoming key participants in chronic rejection [13].

In antitumor immunity, the same cell subsets recognize tumor antigens, for example, AFP and GPC3, via the T cell receptor (TCR) and exert specific cytotoxicity against HCC cells [14]. Expression of inhibitory molecules like PD-L1 in the tumor microenvironment (TME) can induce CTLs exhaustion, which immune checkpoint inhibitors (ICIs) aim to reverse [15]. Furthermore, functional subsets like T follicular helper cells exhibit divergent roles depending on context: they can drive the formation of transplantation-associated tertiary lymphoid structures and rejection [16], but may also assist in antitumor antibody production. Therefore, non-selective T cell suppression strategies, while controlling rejection, inevitably weaken antitumor immunity, highlighting the necessity for precise regulation of alloreactivity.

### 2.2. B Lymphocytes and Antibody Responses: From Pathological Damage to Physiological Defense

B cell responses exhibit dual characteristics in the pathophysiology of transplant rejection and tumor immunity. Activated B cells can differentiate into plasma cells producing donor-specific antibodies (DSA), which mediate antibody-mediated rejection through mechanisms including complement activation, Fc receptor-mediated recruitment of inflammatory cells, and endothelial cell activation [17,18]. In contrast, in antitumor immunity, B cells and their produced antibodies play a protective role. Studies show that tumor-infiltrating B cells are associated with improved prognosis and T cell activation in HCC patients [19,20], potentially inhibiting tumor growth through mechanisms like antibody-dependent cellular cytotoxicity, antigen presentation, and pro-inflammatory cytokine secretion [19].

Notably, specific subsets like regulatory B cells express PD-L1 and secrete inhibitory cytokines such as IL-10 and TGF-β, participating not only in transplant tolerance regulation but also suppressing antitumor CTLs responses and promoting immune escape [21,22,23].

These findings indicate that therapeutic strategies targeting B cell responses must move beyond global suppression, focusing instead on specifically eliminating pathogenic subsets, like DSA-producing cells, while preserving or enhancing protective subsets, such as antitumor B cells.

### 2.3. Innate Immune Cells: Shapers of the Microenvironment

Innate immune cells serve as a bridge connecting innate and adaptive immunity, profoundly influencing rejection and antitumor processes by shaping the local immune microenvironment.

Monocytes/Macrophages: In rejection, recipient-derived monocytes infiltrate the graft and differentiate into inflammatory macrophages, secreting pro-inflammatory cytokines and activating T cell responses. Recent research highlights the critical role of intermediate monocytes characterized by CD14^++^CD16^+^ expression in acute rejection: this population highly expresses molecules related to antigen presentation and promotes T cell proliferation directly by secreting resistin binding to the CAP1 receptor on T cells, driving the rejection process [24]. In tumor immunity, macrophages polarize into either antitumor M1 phenotypes or pro-tumor M2 phenotypes (tumor-associated macrophages, TAMs). TAMs in the HCC TME often exhibit an M2-like phenotype, supporting disease progression by inhibiting T cell function, promoting angiogenesis, and facilitating tumor metastasis [25,26]. Targeting strategies like CSF1R inhibition aim to remodel TAMs polarization, representing an important anticancer research direction [27].

Dendritic Cells (DCs): DCs are crucial for activating T cell responses. In rejection, both donor and recipient DCs efficiently present alloantigens, initiating the activation of alloreactive T cells [11,28]. In antitumor immunity, different DC subsets (cDC1, cDC2, LAMP3^+^ DCs) are highly heterogeneous, either promoting CD8^+^ T cell activation or being associated with T cell exhaustion [29,30], with their balance determining the strength of the antitumor immune response.

Natural Killer (NK) Cells: NK cells can kill target cells without prior sensitization. In rejection, they can participate in graft damage via “bystander activation” mechanisms [31]; in antitumor immunity, they are important effector cells for eliminating HCC cells, mediated through activating receptors like NKG2D and NKp30 [32]. However, the HCC TME can lead to impaired NK cell function and reduced numbers, promoting immune escape [33,34].

Immunosuppressive Cell Populations: Myeloid-derived suppressor cells (MDSCs), regulatory T cells (Tregs), and tolerogenic neutrophils are significantly enriched in both the transplanted liver and the HCC TME. They establish a local immunosuppressive microenvironment by secreting mediators like TGF-β, IL-10, and arginase, which not only protect the graft from effector cell attack but also severely weaken antitumor immune surveillance, creating favorable conditions for tumor recurrence [35,36,37].

**Figure 1:**
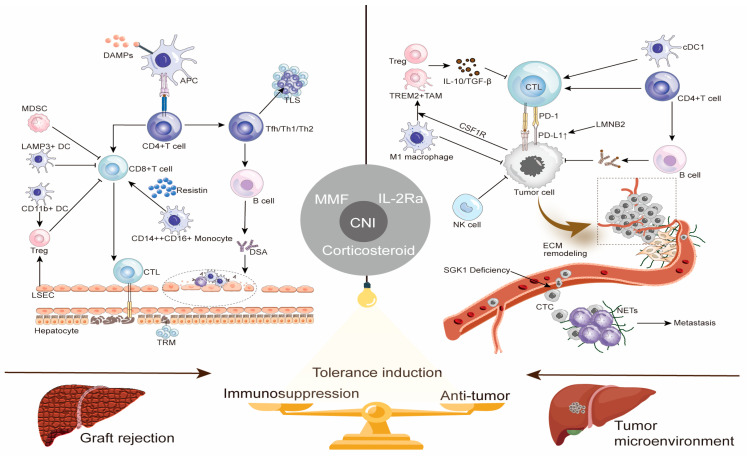
Immunological mechanisms of anti-tumor microenvironment and graft rejection: targets for balancing antitumor immunity and graft tolerance. This schematic illustrates the key cellular and molecular players, as well as potential therapeutic targets, at the crossroads of graft rejection and antitumor immunity: A. Graft Rejection (Left Panel): Initiating Event: Ischemia–reperfusion injury releases DAMPs. Adaptive Immune Activation: APCs (e.g., DCs) activate CD4^+^ T cells, which help B cells produce DSAs (mediating humoral rejection) and activate CTLs (mediating cellular rejection). Chronicity & Regulation: Effector CD8^+^ T cells can form TRMs, mediating chronic rejection. Their activity is modulated by resistin from intermediate monocytes and is negatively regulated by MDSCs, LAMP3^+^ DCs, and Tregs. Liver sinusoidal endothelial cells (LSECs) and CD11b^+^ DCs promote Tregs recruitment. B. Tumor Microenvironment (Right Panel): Primary Anti-tumor Response: CD8^+^ CTLs recognize tumor antigens via TCR, a process inhibited by the PD-1/PD-L1 axis. Secondary Mechanisms & Suppression: Includes humoral immunity and NK cell cytotoxicity. CTLs function is supported by CD4^+^ T cells and cDC1 but suppressed by Tregs and TAMs (via IL-10/TGF-β). Tumor Immune Escape: Tumor cells upregulate PD-L1 via LMNB2 and polarize macrophages to M2 via CSF1R. ECM remodeling in the TIME and SGK1 loss in CTCs promote metastasis, aided by neutrophil extracellular traps. C. Strategies for Immune Balance (Middle): Current Dilemma: Broad CNI-based regimens suppress rejection but promote tumorigenesis. Future Goal: Precise strategies are needed to selectively inhibit alloreactivity while preserving antitumor immunity, aiming for operational tolerance.

To visually illustrate the core roles of the aforementioned immune cells in graft rejection and antitumor immunity, as well as potential regulatory targets, Figure 1 summarizes the key immunological mechanisms of both processes and strategies for achieving immune balance.

### 2.4. Summary: Towards Context-Dependent Precision Immune Regulation

The roles of immune cells in transplant rejection and antitumor surveillance are highly dependent on contextual signals. Current widely used immunosuppressants like calcineurin inhibitors (CNIs), due to their non-specificity, severely damage antitumor immunity while suppressing rejection, placing these recipients at increased risk of recurrence. Breaking this dilemma requires a shift from “global immunosuppression” to “context-dependent precision immune regulation,” i.e., developing innovative strategies that can selectively inhibit alloreactivity, for example, by targeting the intermediate monocyte-resistin axis [24], without affecting antitumor immunity, such as by preserving CTLs and NK cell function. This demands a deeper understanding of the regulatory networks controlling cell function in different immune environments, thereby providing new pathways to achieve the dual goals of long-term graft survival and effective tumor control.

## 3. The Unique Immune Microenvironment of the Liver and Hepatocellular Carcinoma

The liver is an organ with unique immune properties. Its inherent immune-tolerant microenvironment provides relatively favorable conditions for liver transplantation but also creates space for the occurrence and immune escape of HCC. A deep understanding of the formation mechanisms of this microenvironment and its dual role in transplantation and tumors is fundamental to developing highly effective therapeutic strategies.

### 3.1. The Liver’s Innately Tolerant Immune Environment

The establishment of the liver’s immune tolerant state relies on the synergistic action of various resident cells [38]. Although not professional APCs, hepatocytes can present antigen in a way that leads to antigen-specific T cell anergy or apoptosis via low expression of costimulatory molecules like CD80 and CD86 and high expression of PD-L1. Liver DCs are typically immature, secrete IL-10, and induce Tregs differentiation. Kupffer cells, as liver-resident macrophages, effectively suppress T cell responses by producing inhibitory cytokines like IL-10 and TGF-β. Liver sinusoidal endothelial cells (LSECs) also present antigen in a non-costimulatory manner and induce Tregs generation. Furthermore, the naturally high proportion of Tregs within the liver further consolidates the local immunosuppressive state.

Single-cell transcriptomic studies provide new evidence for this tolerogenic process. Research found that myeloid cell populations, such as Kupffer cells and CD11b^+^ dendritic cells, within liver allografts highly express anti-inflammatory factors like IL-10 early after transplantation, actively suppressing cytotoxic T cell function and promoting the recruitment and activation of Tregs, thereby rapidly establishing an inhibitory microenvironment conducive to graft survival [39].

More importantly, the liver’s immune tolerance property is transforming from a passive physiological phenomenon into a therapeutic domain that can be actively targeted. Emerging research strategies aim to precisely utilize the inherent immunoregulatory functions of liver non-parenchymal cells, such as LSECs, to actively induce antigen-specific immune tolerance by delivering specific antigens to them [40]. This provides novel ideas for manipulating the local liver immune environment to achieve therapeutic goals.

### 3.2. The Dual Impact of Immune Tolerance: Graft Protection vs. Tumor Escape

The liver’s inherent immune tolerance is a “double-edged sword,” having distinctly different clinical impacts on liver transplant recipients. On one hand, this property makes the liver an “immunologically privileged” organ, with a significantly lower incidence of chronic rejection after liver transplantation compared to other solid organ transplants [41]. The most compelling evidence is the phenomenon of “operational tolerance” (OT) after liver transplantation, where approximately 20% of recipients maintain normal graft function after complete cessation of immunosuppressants [42]. Achieving OT not only spares patients the toxic side effects of lifelong medication but also shows long-term prognosis comparable to those on continuous immunosuppressive therapy [43]. Notably, the OT state can be maintained even in the presence of DSA [44], highlighting its clinical feasibility and application value.

On the other hand, this same immunosuppressive microenvironment provides a breeding ground for HCC growth and immune escape. Studies indicate that this physiological immune tolerance is a key mechanism for HCC resistance to immunotherapy [15]. Crucially, this immunosuppressive microenvironment is not static but is profoundly and dynamically regulated by the “gut–liver axis.” Recent research has found that gut microbiota and their metabolites remotely regulate the liver’s immune landscape through this axis. As exemplified by the work of Hu et al., who found significantly reduced Lactobacillus reuteri and acetate levels in HCC mice, leading to increased IL-17A secretion by type 3 innate lymphoid cells, thereby promoting tumor progression [45]. This provides a new “immunometabolic” perspective for understanding the formation of the HCC immunosuppressive microenvironment, suggesting that immune-metabolic signals originating from the gut can remotely regulate immune cell function within the liver, influencing tumor progression.

Consequently, therapeutic strategies for HCC recipients after liver transplantation need to be exceptionally refined, aiming to break local tumor immune tolerance without triggering systemic autoimmunity or graft rejection.

### 3.3. Immune Microenvironment Characteristics of HCC and Its Metastases

The tumor immune microenvironment of HCC is a highly heterogeneous, dynamically evolving, and immunosuppressive ecosystem. Its features include abnormal angiogenesis, a persistent inflammatory state, and extracellular matrix remodeling [46]. Within this system, various immunosuppressive cells, including Tregs, MDSCs, and M2-type TAMs, collectively induce effector T cell exhaustion by secreting cytokines like IL-10 and TGF-β, and by highly expressing immune checkpoint molecules such as PD-1, T-cell immunoglobulin and mucin-domain containing-3 (Tim-3), and Lymphocyte-activation gene 3 (LAG-3) [47].

Tumor cells themselves actively participate in immunosuppression through various intrinsic mechanisms. A recent study found that Lamin B2 (LMNB2) can act as a transcription factor directly binding to the promoter region of PD-L1 and driving its transcription. This LMNB2-PD-L1 signaling axis operates independently of the classical interferon signaling pathway, providing a novel mechanistic explanation for how HCC cells actively upregulate immune checkpoints to evade immune surveillance [48]. Single-cell studies further categorize HCC cancer cells into functional prototypes: metabolic (APOEhi), stem-like (CD44hi), and inflammatory (SAA1/2hi). Among these, metabolic-type cells drive the polarization of TREM2^+^ macrophages via secreted oxidized low-density lipoprotein, collaboratively constructing an immune-suppressive niche that excludes CD8^+^ T cells, thereby mediating resistance to immunotherapy [49].

The immune microenvironment of HCC lung metastasis exhibits unique heterogeneity distinct from the primary site, serving as a “soil” suitable for tumor colonization and growth [35]. Circulating tumor cells (CTCs) are the seeds of metastasis, and their immune escape capability is crucial. Research found that loss of serum/glucocorticoid regulated kinase 1 (SGK1) expression in CTCs promotes metastatic colonization through a key mechanism: SGK1 deficiency weakens CD8^+^ T cell-mediated, RIPK1-dependent necroptosis—a crucial cell death pathway limiting metastasis [50]. This allows CTCs to survive immune attack. Additionally, successfully colonized SGK1-deficient cells further shape the metastatic microenvironment, inducing CD8^+^ T cell differentiation towards a terminally exhausted phenotype, forming an immune-suppressive niche favorable for metastatic growth [50]. Neutrophil extracellular traps have also been found to promote CTC entrapment and metastasis [51].

Immunosuppressant use post-liver transplantation profoundly reshapes the metastatic microenvironment. Importantly, different categories of immunosuppressant may have diametrically opposite effects on metastasis. CNIs are reported to promote lung metastasis by facilitating M2 macrophage polarization, suppressing dendritic cell function, and enhancing epithelial–mesenchymal transition (EMT), whereas mTOR inhibitors may inhibit metastasis by enhancing M1 polarization, promoting antigen presentation, and suppressing EMT [35]. This highlights that for HCC patients after liver transplantation, choosing an immunosuppressive regimen with antitumor properties is crucial for controlling metastasis.

Overall, the immune microenvironment of HCC and its metastases is a dynamic network shaped by tumor cells, various immune cells, stromal cells, and external interventions, like immunosuppressants. Precise targeting of its key nodes, for instance, the SGK1-RIPK1-necroptosis axis, the LMNB2-PD-L1 axis, or specific immune cell subsets, is a new direction for overcoming immunotherapy resistance and inhibiting tumor metastasis in the future.

## 4. Impact of Standard Immunosuppressive Regimens on HCC Antitumor Immunity and Recurrence

A CNI-based triple immunosuppressive regimen is routinely used after liver transplantation. Nonetheless, intensified immunosuppression is significantly associated with increased tumor recurrence risk, with the core contradiction being that non-specific immunosuppression protects the graft while broadly weakening the body’s antitumor immune surveillance function [52]. Currently, there is a lack of standardized management strategies for effectively balancing the prevention of rejection with the preservation of antitumor immunity.

### 4.1. CNIs: The Dual Challenge of Efficacy and Risk

CNIs efficiently inhibit T cell activation and proliferation by inhibiting calcineurin downstream of the TCR, blocking NFAT nuclear translocation and the transcription of key cytokines like IL-2, thus forming the cornerstone of immunosuppressive therapy [53,54,55]. Additionally, CNIs can also indirectly attenuate B cell-mediated antibody responses and regulate costimulatory molecule expression, further enhancing immunosuppressive effects [35,56].

Furthermore, this potent suppression extends to their regulation of innate immune cells. Under conditions mimicking clinical drug exposure levels in vitro, Qin et al. demonstrated that both tacrolimus and cyclosporine significantly inhibited NK cell secretion of IFN-γ and expression of the degranulation marker CD107a [57]. Given that NK cells are a crucial early source of IFN-γ, this effect may indirectly attenuate T cell activation, further explaining the central role of CNIs in preventing rejection. However, the same study found that CNIs had a limited impact on NK cell-mediated direct tumor cell killing activity [57]. This suggests that while potently suppressing allogeneic T cell responses, CNIs may relatively preserve some of the anti-tumor functions of NK cells, revealing the complexity of their mechanism of action. Single-cell studies further illuminate the extensive remodeling of the intrahepatic immune landscape by CNIs in vivo: tacrolimus-based immunosuppression significantly alters the proportions and states of innate immune cells in the transplanted liver, including monocyte/macrophage subsets and neutrophil populations [58]. This systemic suppression of the immune microenvironment provides an important cellular-level explanation for why CNIs, while effective in preventing rejection, increase the risk of HCC recurrence.

Multiple studies have confirmed that CNIs are independent risk factors for HCC recurrence after liver transplantation [56]. Their pro-tumor mechanisms involve multiple levels: (1) Direct pro-tumor effects: CNIs can enhance tumor angiogenesis and invasiveness by upregulating IFI27/VEGFA [59], and activate proto-oncogenes and enhance TGF-β expression in a dose-dependent manner, thereby promoting recurrence and metastasis [60]. (2) Epigenetic regulation: Tacrolimus can inhibit m6A modification of lncRNA, upregulating CCL2 and CD47 expression, promoting TAM infiltration and inhibiting their phagocytic function, thus remodeling the immunosuppressive microenvironment [61]. (3) Promoting metastasis: CNIs induce EMT through activating the TGF-β/Smad pathway, increasing CTC numbers, and accelerating tumor progression [35,62,63].

Notably, the inhibition of Tregs by CNIs [64] may seem beneficial, but the overall broad suppression of effector T cells is ultimately detrimental to tumor control. Furthermore, the nephrotoxicity of CNIs also drives changes in therapeutic strategy. Studies show that early CNI withdrawal or conversion to a CNI-free regimen, for instance, using everolimus combined with mycophenolate mofetil, can significantly improve renal function, as shown by an eGFR of 95.8 versus 76.0 mL/min/1.73 m^2^ [65], but requires vigilance for a corresponding increase in rejection risk.

CNI exposure levels are closely related to recurrence risk. Tacrolimus trough levels > 10 ng/mL or cyclosporine trough levels > 300 ng/mL can triple the recurrence risk, especially during the first month postoperatively [52]. Recent research further emphasizes that cumulative exposure to tacrolimus (CET) is a more precise indicator for predicting malignancy risk; every 20% increase in CET within the first 3 months post-transplant increases HCC recurrence risk by 26% [66]. This suggests the systemic immunosuppressive effect of CNIs is dose-dependent, and CNI minimization or withdrawal has become an important clinical strategy, albeit one requiring individualized weighing of rejection versus tumor recurrence risks.

### 4.2. Antimetabolites: The Potential Advantages of Mycophenolate Mofetil

Mycophenolate mofetil (MMF) selectively blocks T and B lymphocyte proliferation by inhibiting purine synthesis [67,68]. Notably, its active component, mycophenolic acid, at clinical concentrations, can upregulate p53 expression, induce S-phase arrest in HCC cells, and downregulate VEGF to inhibit tumor angiogenesis, demonstrating potential antitumor effects [69]. However, the immunosuppressive effects of MMF are not limited to adaptive immunity. The study by Qin et al. revealed that at clinically effective plasma concentrations, MMF impairs the cytotoxic activity of NK cells. More importantly, transcriptomic analysis found that NK cells treated with MMF significantly upregulated the expression of inhibitory receptors Siglec-7 and Siglec-9 [57]. These receptors are negative regulatory checkpoints for NK cell function, and their upregulation may weaken NK cell immune surveillance capacity, potentially partially offsetting its direct anti-tumor effects and introducing uncertainty for tumor management in liver transplant recipients.

MMF pharmacokinetics show significant individual variation, making therapeutic drug monitoring (TDM)-guided personalized dosing crucial. Research supports maintaining the area under the concentration-time curve (AUC) of mycophenolic acid over 0–12 h within the therapeutic range of 30–60 mg·h·L^−1^ [70]. A multicenter randomized trial confirmed that TDM-guided MMF dosing, targeting an AUC of 45 mg·h/L, can effectively support a steroid-free immunosuppression regimen, with an acute rejection rate of 8% that was non-inferior to steroid-containing regimens [70], providing a treatment option balancing safety and efficacy for HCC LT recipients.

### 4.3. Corticosteroids and IL-2 Receptor Antagonists

Corticosteroids exert non-specific anti-inflammatory effects by broadly inhibiting cytokine expression and T cell proliferation [71], but they can promote Tregs expansion and MDSCs recruitment, impair antitumor immune surveillance, and potentially increase HCC recurrence risk [72]. Simultaneously, in vitro studies show that compared to other classes of immunosuppressants, corticosteroids exhibit particularly strong inhibition of NK cell cytotoxic activity and IFN-γ secretion [57], which aligns with the observed higher recurrence risk in liver transplant recipients associated with their use.

Basiliximab, an IL-2 receptor antagonist, inhibits T cell activation by blocking the IL-2Rα chain, often used for induction therapy to delay CNI initiation or avoid steroid use [73,74,75], benefiting renal function protection [76]. Its impact on tumor prognosis remains controversial, with some studies reporting a potential increase in recurrence risk [77], but recent large studies show that non-depleting induction therapy, including the use of IL-2 receptor antagonists, does not worsen tumor recurrence [78].

**Figure 2:**
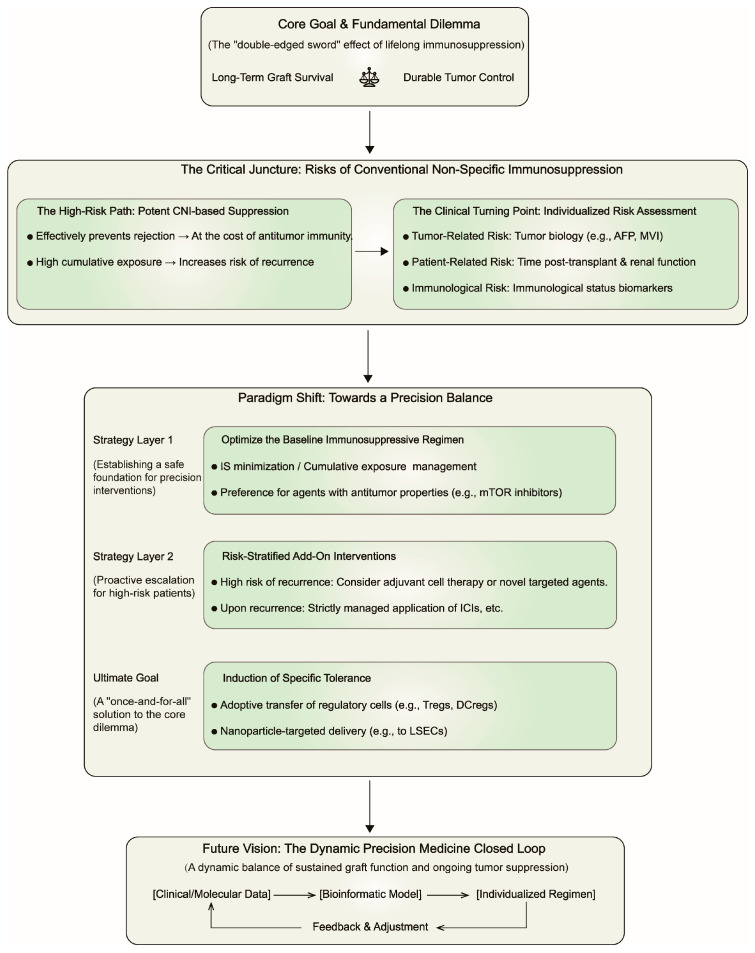
A decision framework for precision immunological management of HCC after liver transplantation. The framework outlines the transition from the dilemma of conventional immunosuppression (top) to a paradigm of precision balance. This is achieved through a two-layer strategy of baseline regimen optimization and risk-stratified interventions, guided by individualized risk assessment, with the ultimate goal of inducing operational tolerance. The closed-loop at the bottom depicts the vision for dynamic precision medicine. Abbreviations: CNI, calcineurin inhibitor; MVI, microvascular invasion.IS, immunosuppression. ICIs, Immune checkpoint inhibitors.

Collectively, although CNIs are the primary risk factors for HCC recurrence, all immunosuppressants contribute to the overall immunosuppressive burden. Hence, regimen optimization must extend beyond adjustments of single drugs to encompass comprehensive strategies including CNI minimization, steroid avoidance/withdrawal, and a preference for drugs with potential antitumor properties like MMF. We propose a decision-making framework (Figure 2) for guiding post-LT precision immunotherapy, which pivots from non-specific immunosuppression to a dynamically balanced, risk-stratified paradigm.

## 5. Strategies for Balancing Anti-Rejection and Anti-Tumor Effects

### 5.1. Immunosuppression Minimization and Withdrawal Regimens

#### 5.1.1. CNI Minimization, Conversion, and Cumulative Exposure Management

The core goal of CNI minimization strategies is to reduce their nephrotoxicity, metabolic diseases, and tumor recurrence risk while maintaining sufficient immunosuppressive efficacy to prevent rejection [79]. The clinical concept of CNI minimization has a history spanning over four decades. The initial driving force for this strategy was primarily to mitigate the inherent side effects of CNIs, such as nephrotoxicity and neurotoxicity. This thinking was reflected in early immunosuppressive protocols, for example, utilizing induction therapy—by providing potent immune coverage during the initial transplant period to create a window for reducing CNI dosage. A landmark study in 1996 demonstrated that immunoprophylaxis with a monoclonal antibody against the IL-2 receptor α chain (CD25; BT563) effectively prevented acute rejection without increasing the risk of infection, offering a new pathway for achieving effective early immunosuppression within cyclosporine-based therapy [80]. Such studies laid the theoretical and practical foundation for combination therapies aimed at CNI dose reduction or delayed introduction.

However, over the past two decades, with the rise of transplant oncology, substantial evidence has firmly established CNIs as independent risk factors for HCC recurrence after liver transplantation [52,56,66]. Consequently, the current CNI minimization strategy, building upon traditional organ protection, has evolved into a crucial core strategy for oncological management. Prospective randomized controlled trials provide high-level evidence for early CNI minimization combined with mTOR inhibitors. The HEPHAISTOS study confirmed that employing everolimus combined with reduced-dose tacrolimus (target trough level < 5 ng/mL) in the very early post-transplant period (7–21 days post-op) resulted in a composite efficacy endpoint rate (graft loss, death, or biopsy-proven acute rejection) comparable to the standard regimen (7.7% vs. 7.9%), while demonstrating significant potential for renal protection, with greater benefit observed particularly in patients with strict therapeutic drug monitoring [81]. This robustly demonstrates that this strategy can achieve effective tacrolimus reduction without compromising graft protection.

Traditional implementation relies on monitoring trough blood levels. Currently, immunosuppressive management is evolving from reliance on single tacrolimus trough levels toward a precision medicine paradigm utilizing Cumulative Exposure to Tacrolimus (CET). It is calculated as the area under the curve of serial trough concentrations over time, providing a more accurate measure of overall drug exposure and a superior predictor of long-term risks compared to isolated level checks. Large-scale studies have established that the CET within the first 3 months post-transplant is an independent predictor for post-transplant malignancy. Specifically, each 20% increase in the 3-month CET is associated with an 11% higher risk of any cancer (HR 1.11). Notably, the risk of hepatocellular carcinoma recurrence increases significantly by 26% for the same CET increment (OR 1.26) [66]. Therefore, maintaining the CET below specific thresholds—for instance, a 3-month CET of <580 ng·d·mL^−1^, which defines the “minimization” strata—is recommended to mitigate cancer risk [66]. This strategy is particularly crucial for high-risk patients, such as the elderly, active smokers, and those with a history of alcoholic liver disease.

In clinical practice, CNI dose reduction is feasible but complete withdrawal success is limited. Shaked et al.’s randomized trial showed that 67.8% (52/77) of subjects could reduce CNI doses to below 50% of baseline within 1–2 years post-transplant, but only 13% (10/77) achieved complete withdrawal for over 1 year [82]. Successful withdrawal is significantly associated with time post-transplant; a multicenter trial showed withdrawal success rates of 79% in recipients >10.6 years post-transplant compared to only 38% in those >5.7 years post-transplant [83]. However, vigilance is required for the increased risk of rejection associated with complete CNI withdrawal. A large, multicenter, randomized controlled trial, the H2304 study, provides clear cautionary evidence for this. In this study, the tacrolimus elimination arm, where patients were converted to everolimus monotherapy 30 days post-operatively, exhibited a significantly higher incidence of treated biopsy-proven acute rejection (tBPAR) compared to the standard and reduced-dose groups (incidence rates: 16.5%, 7.0%, and 2.9%, respectively), leading the study’s data monitoring committee to prematurely terminate randomization into the tacrolimus elimination arm [84]. This clearly indicates that aggressive CNI withdrawal strategies in the early post-liver transplant period may lead to unacceptable clinical risks.

Therefore, the optimized strategy for oncological considerations is to combine other drugs, such as MMF, during CNI reduction or withdrawal, rather than simple withdrawal. Notably, the clinical outcomes following CNI withdrawal are highly dependent on the timing of withdrawal and the alternative immunosuppressive regimen. The H2304 study showed that early conversion to everolimus monotherapy failed due to high rejection rates [84]. However, the strategy in the same study involving early introduction of everolimus combined with reduced-dose tacrolimus resulted in lower acute rejection rates while effectively preserving renal function; this approach was further corroborated by the HEPHAISTOS study [81]. Conversely, conversion from CNIs to mycophenolate mofetil (MMF) monotherapy demonstrates better feasibility and safety in selected, long-term stable patients post-transplant. A large retrospective study showed that conversion from CNIs to MMF monotherapy in recipients with a median transplant time of 67 months significantly improved renal function with an acute rejection rate of only 7.4% [85]. UK guidelines recommend an MMF dose increase combined with CNI reduction for patients with renal impairment [86].

#### 5.1.2. Steroid-Free Immunosuppression Regimens

The liver’s immune tolerance characteristic provides the basis for early steroid withdrawal in HCC LT recipients. Current clinical practice widely advocates steroid discontinuation within 3–6 months postoperatively [70,78,87].

Substantial evidence supports the feasibility and benefits of this strategy. Compared to steroid maintenance, successful withdrawal eliminates the need to maintain tacrolimus at high levels and significantly reduces tumor recurrence rates and the risk of related adverse effects [87]. A 2018 meta-analysis evaluating randomized clinical trials of steroid avoidance/withdrawal versus steroid-containing regimens confirmed no statistical difference in mortality and graft loss between the groups, but a slightly higher acute rejection rate (RR 1.33) [88]. Data from the China Liver Transplant Registry show that steroid-free immunosuppression allows HCC patients to achieve better survival benefits and fewer adverse reactions, with higher safety in those meeting the Milan criteria [72].

Prospective randomized controlled trials provide higher-level evidence. The research by Saliba et al. [70] demonstrated that a regimen using tacrolimus combined with TDM-guided intensified and individualized adjusted MMF allowed complete avoidance of corticosteroids from postoperative day 1. This strategy resulted in a treated acute rejection rate at 1 year (8%) comparable to the standard steroid-containing regimen (9%), while significantly reducing the incidence of new-onset diabetes (19.8% vs. 32.6%) [70]. This confirms that early steroid avoidance, under effective baseline immunosuppressive protection, provides significant metabolic benefits without increasing rejection risk.

### 5.2. Immunosuppressants with Antitumor Properties: mTOR Inhibitors

mTOR inhibitors, namely sirolimus and everolimus, are unique in liver transplant immunosuppression for providing both immunosuppressive and antitumor dual benefits. Their immunosuppressive action primarily involves blocking mTORC1 complex signaling, affecting T and B lymphocyte proliferation and differentiation, inhibiting dendritic cell maturation, and reducing Tregs production [7,35,89].

Regarding antitumor effects, mTOR signaling pathway activation is associated with poor pathological features, such as poor differentiation and vascular invasion, as well as early recurrence and poor prognosis in HCC [90]. mTOR inhibitors exert antitumor effects through multiple mechanisms: (1) Direct tumor growth inhibition: By downregulating HIF-1α and VEGF expression, they inhibit tumor cell proliferation and angiogenesis [91]. (2) Reshaping the immune microenvironment: They promote macrophage polarization towards the antitumor M1 phenotype [92] and enhance dendritic cell antigen presentation function [93], thereby improving tumor immune surveillance; simultaneously, in the context of mTOR inhibitor-based regimens, NK cells demonstrate a favorable phenotype characterized by reduced expression of inhibitory receptors like NKG2A and enhanced expression of activation markers such as CD107a [57]; compared to CNI-dominated regimens, mTOR inhibitors contribute to a more favorable trend in immune cell population changes, such as modulating pro-inflammatory monocyte subsets [58]. (3) Inhibiting the metastatic process: They have been shown in various models to reduce CTCs numbers [35,94] and inhibit the EMT process by downregulating molecules like EpCAM [35,95].

Clinical research widely supports this dual benefit. A meta-analysis showed that mTOR inhibitor-based regimens significantly reduce early recurrence rates after liver transplantation for HCC and improve recurrence-free survival and overall survival compared to CNI-based regimens [89]. Sirolimus use for more than 3 months is an independent protective factor for improved survival, with benefits particularly significant in patients with high AFP levels and poorer tumor biology [96]. US large transplant cohort data further reveal its tumor-specific advantage: HCC patients receiving sirolimus had a 5-year survival rate as high as 83%, significantly better than other regimens (69%), while no such survival difference was observed in non-HCC recipients [97]. Furthermore, mTOR inhibitor regimens significantly improve CNI-associated renal impairment [98], although attention must be paid to side effects like induced lipid metabolism disorders [99].

Recent evidence also establishes the comprehensive advantage of mTOR inhibitors in reducing non-tumor-related mortality. Studies show they can reduce the risk of non-tumor-related death by 58–74% in liver transplant recipients exceeding Milan/Hangzhou criteria [100], through mechanisms involving direct anti-infection effects, renal protection, and indirect impacts by reducing tumor recurrence. This makes them a strategic choice for balancing graft survival, tumor control, and patient long-term survival risks.

### 5.3. Emerging Immunotherapies in the Transplant Setting

#### 5.3.1. Immune Checkpoint Inhibitors (ICIs): Weighing Risk and Opportunity

ICIs have become an important treatment for advanced HCC, but their use in liver transplant recipients is strictly limited due to the potential to trigger fatal graft rejection [101]. The risk of rejection exists from the perioperative period onward, related to factors such as drug type, washout period, immunosuppression intensity, and graft immunogenicity [102].

Studies on pre-transplant use, specifically as bridging or downstaging therapy, show relatively controllable risks. Existing research has explored the feasibility of ICIs as pre-transplant bridging or downstaging therapy, as summarized in Table 1 [103,104,105,106,107,108,109,110]. As the largest multicenter retrospective study to date by sample size, Guo et al. [106] reported a 27.7% acute rejection rate in 83 patients and clearly identified a time interval between the last ICI administration and liver transplantation (TLAT) of <30 days as an independent risk factor for AR (OR = 0.096, *p* < 0.001), highlighting the central role of TLAT in risk stratification. As the first multicenter prospective study, the VITALITY trial indicated a rejection incidence of approximately 16.3% in patients undergoing liver transplantation after ICI therapy, with a promising 3-year post-transplant survival rate (85%) [103]. This collective evidence points towards the washout period (TLAT) as a key modifiable factor. Current evidence suggests a washout period <30 days significantly increases rejection risk [106,107], while maintaining a washout period of about 3 months is perhaps a core strategy for risk reduction [101].

However, when interpreting these AR incidence data, it is crucial to recognize a significant limitation of current studies: the vast majority of reports fail to elaborate in detail on standardized post-transplant management protocols and universally lack direct comparison with control groups not exposed to ICIs [103,106,107,108]. For instance, although multiple studies mention the use of CNI-based immunosuppressive regimens, systematic therapeutic drug monitoring (TDM) data for drugs like tacrolimus are generally lacking to clarify the association between immunosuppression intensity and rejection events. Notably, the study by Wang et al. provides an instructive case, reporting that the median tacrolimus trough level at the time of rejection was only 7.1 μg/L, and rejection was successfully reversed by increasing the concentration to 15.9 μg/L [109], offering direct evidence for the critical role of TDM in managing such high-risk patients. Furthermore, heterogeneity in post-operative care protocols across multicenter studies makes it difficult to compare AR incidence rates horizontally and determine whether they are attributable to ICIs themselves or differences in post-operative management [106,108]. It is worth mentioning that a recent conference report, using matched controls, preliminarily suggested no significant association between ICI exposure and rejection risk [105], providing a valuable clue and direction for future more in-depth research. However, an excessively long washout period undoubtedly raises concerns about tumor progression, especially in immunotherapy-sensitive individuals, and alternative therapies may lose the benefits of preoperative immunotherapy. A consensus on the optimal washout period has not been fully established, and study results regarding safety thresholds vary. In addition, the exact interval of the washout period may also be influenced by the pharmacokinetics of the specific immunotherapeutic drug, which is currently an empirically imprecise 4- to 6-week time period extrapolated primarily from the pharmacokinetics of commonly used ICI drugs.

Post-transplant use data are associated with higher risk, and general advice is to avoid use in the early postoperative period, especially within the first year [102,111]. Extremely limited studies are underway such as NCT06254248, NCT04564313, NCT03966209. For patients with post-transplant recurrence and no other effective therapies, a very cautious benefit-risk assessment under multidisciplinary guidance is required. Suggested measures include close monitoring of liver function, testing graft PD-L1 expression—as the risk may be relatively lower in PD-L1 negative grafts—and maintaining or intensifying baseline immunosuppression during use [102,112].

Future development directions lie in precision individualized treatment. Studies found that CD8^+^ TRMs infiltrating rejecting liver tissue highly express molecules like PD-1, CTLA-4 [13,101], potentially being the key cellular targets for ICI-induced rejection. Simultaneously, a deeper understanding of tumor molecular subtypes, for example, those where high LMNB2 expression drives PD-L1 upregulation [48], also helps screen patient populations most likely to benefit from ICI therapy with relatively controllable rejection risks, enabling true risk-stratified management.

#### 5.3.2. Adoptive Cell Therapy (ACT)

ACT involves the ex vivo expansion and reinfusion of immune cells with antitumor activity, such as CAR-T, TCR-T, TILs, and CIK cells, to target and eliminate HCC [111]. The core challenge for its application in the liver transplantation field is ensuring tumor specificity, avoiding cross-recognition of graft alloantigens which could trigger rejection.

Among various ACTs, CIK cell therapy has been relatively more studied, with the most extensive safety data in transplant recipients. Studies show that adjuvant CIK therapy after liver transplantation is well-tolerated, without an observed significant increase in rejection, and demonstrates a trend towards improved survival [113]. Allogeneic NK cell infusion therapy also shows good safety and potential recurrence prevention effects [114]. However, data on more precise technologies like CAR-T and TCR-T therapy in liver transplant recipients are scarce; while theoretically more specific, they also carry risks of cytokine release syndrome and off-target toxicity, requiring extreme clinical caution and fine-tuning of systemic immunosuppression regimens during treatment [115,116,117,118,119,120]. Overall, ACT is a highly individualized strategy requiring precise coordination with immunosuppressive regimens.

#### 5.3.3. Gene Therapy and Oncolytic Viruses

These emerging therapies, including miR-22-based gene regulation and the oncolytic virus VG161, exert antitumor effects by directly acting on tumor cells or the local microenvironment, with mechanisms differing from systemic T cell activation by ICIs [121,122,123]. For example, oncolytic viruses specifically replicate in and lyse tumor cells, releasing tumor antigens, thereby initiating an antitumor immune response in situ, acting via an “in situ vaccine” effect, without complete reliance on host naive T cell function [124]. This characteristic makes them theoretically very suitable for liver transplant recipients requiring maintained systemic immunosuppression, offering another promising approach to balancing anti-rejection and antitumor needs. Their synergistic effects with traditional treatments, such as targeted drugs, are also worth exploring in the transplant context.

**Figure 3:**
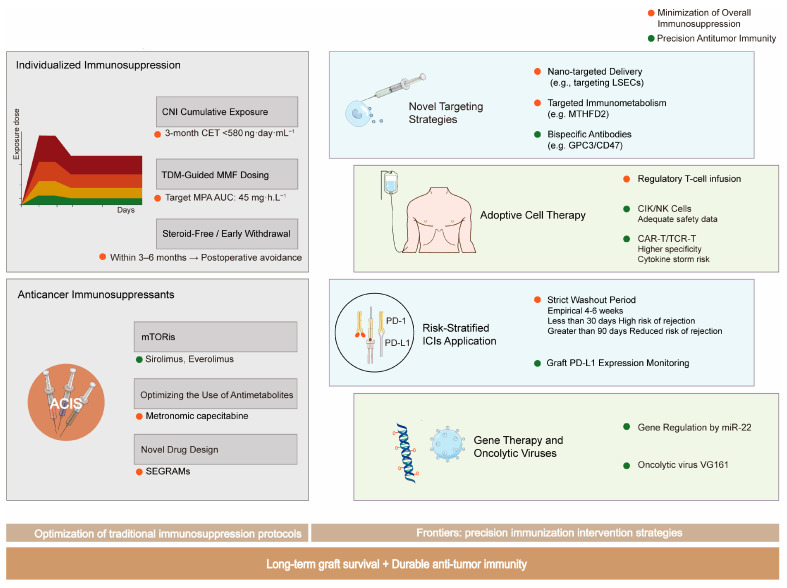
Evolving strategies to balance graft protection and antitumor immunity in liver transplant recipients with hepatocellular carcinoma. A conceptual framework of precision management strategies for hepatocellular carcinoma in liver transplant recipients. The paradigm is shifting from non-specific immunosuppression towards integrated approaches that concurrently ensure long-term graft survival and durable antitumor immunity. Key strategies include (1) Individualized Immunosuppression, focusing on managing calcineurin inhibitor (CNI) cumulative exposure (CET), therapeutic drug monitoring (TDM)-guided dosing of mycophenolate mofetil (MMF), and steroid avoidance; (2) Leveraging Anticancer Immunosuppressants, such as mTOR inhibitors; and (3) Novel Precision Immuno-interventions, including risk-stratified use of immune checkpoint inhibitors (ICIs) with graft PD-L1 monitoring, adoptive cell therapies, and emerging approaches like bispecific antibodies and nano-targeted delivery.

A comprehensive overview of the evolving strategies to achieve the critical balance between preventing allograft rejection and preserving antitumor immunity is summarized in Figure 3.

### 5.4. Immune Tolerance Induction: The Ultimate Goal and Current Strategies

Immune tolerance induction is the ultimate goal in liver transplantation, aiming to achieve complete cessation of immunosuppressant, thereby resolving the contradiction between immunosuppression and antitumor function once and for all. Current strategies, though not yet mature, represent the future direction.

Adoptive Regulatory Cell Infusion: Infusion of ex vivo expanded Tregs or regulatory DCs has shown potential to induce operational tolerance in early clinical studies, but their efficacy stability, cell source, and long-term safety still require optimization [125,126,127,128].

Nanoparticle-Targeted Delivery is an innovative strategy beyond cell infusion, using engineered nanoparticles to precisely deliver disease-specific antigens to inherent tolerogenic antigen-presenting cells like LSECs, thereby actively “reprogramming” the immune system to induce antigen-specific immune tolerance [40]. This method is more scalable and controllable. A variety of innovative cellular, genetic and pharmacological-based strategies have entered clinical testing aimed at proactively inducing immune tolerance. As shown in Table 2, these studies cover a diverse range of pathways from infusion of regulatory cells, genetic engineering of modified cells or organs, to utilization of drug combinations.

Despite the immense challenges, these strategies aiming to precisely regulate specific immune pathways rather than broadly suppress the immune system fundamentally align with the theme of this review and are core directions for achieving individualized and refined immune management in the future.

## 6. Future Directions and Research Priorities

The field of liver transplantation is undergoing a critical shift from non-specific immunosuppression towards precision targeted therapy. Future research will focus on translating basic research findings into clinical practice, developing innovative strategies that can selectively inhibit rejection while preserving antitumor immunity.

### 6.1. Developing New Mechanism-Based Selective Therapeutic Strategies

#### 6.1.1. Traditional Drug Optimization and New Drug Design

Inspired by the dual benefits of mTOR inhibitors, “anticancer immunosuppressants” with both antitumor and immunosuppressive effects have become a research hotspot [53]. These include optimized use of classic antimetabolites, as exemplified by metronomic capecitabine which can prevent recurrence post-liver transplantation without causing acute rejection [129], and novel drug designs. For instance, novel selective glucocorticoid receptor modulators can precisely modulate GR conformation, successfully dissociating anti-inflammatory effects from metabolic side effects. The clinical candidate drug GRM-01 induced a mere 14.0 ± 6.4% tyrosine aminotransferase expression in vitro, compared to 92.4 ± 5.3% with prednisone, and inhibited osteoprotegerin by 58 ± 16%, whereas prednisone mediated complete inhibition [130], providing new directions for traditional drug reform.

#### 6.1.2. Novel Strategies Targeting Specific Rejection Pathways

Precise intervention in key signaling pathways of transplant rejection shows great potential. The discovery of the resistin (Retn)-CAP1 signaling axis provides a specific target for developing novel anti-rejection therapies [24]. The AU-siRetn nanotherapy developed based on this efficiently silences resistin expression, significantly alleviating rejection in rat models without significant toxicity, offering a new approach for precisely targeting rejection pathways while avoiding global immunosuppression [24].

Bispecific antibodies represent another important direction. The GPC3/CD47 bispecific antibody co-targets a tumor antigen and the “don’t eat me” signal, namely the CD47–SIRPα pathway. It effectively circumvents the toxicity of traditional anti-CD47 monoclonal antibodies and significantly extends its serum half-life to approximately 13.4 days from approximately 2.4 days [131]. In HCC models, this bispecific antibody showed efficacy significantly superior to single or combination antibody therapy [131], providing a new strategy for preventing post-transplant recurrence.

#### 6.1.3. Innovative Methods Targeting Immunometabolism

Immunometabolic reprogramming offers new targets for precise intervention. Studies show that the mitochondrial one-carbon metabolism enzyme MTHFD2 is a key metabolic checkpoint driving the differentiation of pathogenic T follicular helper cells [16]. Due to its low expression in quiescent somatic cells, specific inhibition of MTHFD2 can effectively curb pathological immune responses with a good potential safety profile, providing a new pathway for selectively inhibiting rejection while preserving antitumor immunity.

### 6.2. Biomarker-Driven Individualized Therapy

#### 6.2.1. Development and Application of Novel Biomarkers

Achieving precision medicine requires reliable biomarkers to guide clinical decision-making. CTCs, circulating tumor DNA, and exosomal contents are crucial for early detection of HCC metastasis [49]. Recent research found that the transcriptomic profile of peripheral blood mononuclear cells in transplant recipients is highly correlated with the immune status within the graft liver [16]; monitoring changes in specific immune cell subsets or functional gene expression can non-invasively assess graft status, providing real-time guidance for adjusting immunosuppressive regimens.

CET, as an emerging quantification tool, has been confirmed to be significantly associated with renal injury and malignancy risk [66]. Integrating it into clinical decision systems enables real-time dynamic risk assessment of tacrolimus exposure, providing an objective basis for individualized immunosuppressive minimization.

TDM remains an important strategy for precise dosing. mycophenolic acid exposure (AUC0–12 h) and adjusting MMF dose accordingly can significantly improve patient exposure, supporting more aggressive minimization strategies, such as safe steroid withdrawal [70].

#### 6.2.2. Challenges and Prospects in Predicting Operational Tolerance

Research on predictive biomarkers for OT has made progress but still faces significant challenges. Current studies show that CD4^+^CD25^+^FOXP3^+^ Tregs may play a role in OT establishment; tolerant patients often have Tregs enrichment in the graft, and peripheral blood FOXP3 expression is also correlated with tolerance status [132,133,134]. Nevertheless, Tregs have clear limitations as predictive biomarkers: their frequency and counts are highly dynamic, showing instability in longitudinal assessments before and after immunosuppressant withdrawal [132], and some definitively diagnosed OT patients did not show significant Tregs enrichment in grafts, suggesting the OT state may not completely depend on this cell population [135].

Gene expression profiling shows better predictive potential. Research found that pre-withdrawal whole blood gene expression signatures might non-invasively predict successful mTOR inhibitor withdrawal [136]. Notably, some previously reported biomarkers, including iron homeostasis-related genes, failed validation in multicenter trials [137,138], highlighting the importance of multicenter studies in biomarker validation.

At the mechanistic level, microenvironmental features like increased donor-specific T cell senescence, intragraft C4d deposition, and elevated immune synapse density may serve as candidate OT biomarkers [139]. Specific T cell subsets, such as activated CD4^+^ T cells and highly differentiated alloreactive CD4^+^ T cells, have also been confirmed to potentially distinguish OT recipients [140]. However, changes in donor-reactive T cells are a common phenomenon after liver transplantation, not unique to OT, and not necessarily related to the success or failure of early immunosuppressant withdrawal.

Clinical practice must pay special attention that approximately 20–30% of OT patients still have histologically active alloimmune fibroinflammatory lesions [138], so graft injury must be assessed by biopsy before immunosuppressant withdrawal [141]. Future research should focus on developing multi-parameter combined prediction models, integrating immune cell monitoring, gene expression profiles, and clinical indicators, utilizing single-cell sequencing technology and artificial intelligence algorithms to achieve precise guidance for individualized immunosuppressant withdrawal strategies.

#### 6.2.3. Translational Opportunities from Bench to Bedside

The latest basic research provides multiple translational directions for clinical practice: developing drugs targeting the resistin pathway, guiding individualized treatment strategies by SGK1 expression, exploring combination therapies of LMNB2 inhibitors and immune checkpoint inhibitors, etc. Rooted in a deeper understanding of disease mechanisms, these innovative strategies are expected to achieve true precision medicine.

Building on the deep understanding of liver immune microenvironment regulation mechanisms, such as the ‘gut–liver axis’ [45], gut microbiota modulation represents another promising direction. Studies show that intervening in the ‘gut–liver axis’ through probiotics, prebiotics, or specific metabolites like acetate can selectively regulate the liver immune microenvironment [45]. This strategy may specifically weaken tumor-promoting factors without causing broad immunosuppression, offering a novel, microecology-targeted treatment option for liver transplant recipients.

## 7. Conclusions

The core management of HCC in liver transplant recipients lies in resolving a fundamental immunological contradiction: effectively preventing allograft rejection while maximally preserving and even enhancing the host’s immune surveillance capability against tumors. Traditional broad-spectrum immunosuppressive regimens, particularly high-dose CNIs, non-specifically widely inhibit T lymphocyte function; while protecting the graft, they have been confirmed as important risk factors promoting HCC recurrence through multiple mechanisms including direct pro-tumor effects, epigenetic regulation, and induction of EMT.

Currently, clinical practice is moving from mere immunosuppression intensity control towards a new paradigm centered on precision and balance. This shift is reflected at multiple levels: strategically, evolving from monitoring based on trough blood levels to the precise management of CET, and comprehensively applying CNI minimization/conversion, early steroid-free protocols, and preferential use of mTOR inhibitors with antitumor properties; mechanistically, shifting from global suppression to targeting specific pathogenic pathways, such as the resistin-CAP1 axis, and cell subsets, like intermediate monocytes; therapeutically, cautiously exploring the application window for ICIs and ACT under strict management, and focusing on the ultimate goal of immune tolerance induction.

However, challenges remain. The risk of rejection triggered by immunotherapy, the vast immune heterogeneity among individuals, and the lack of prospective high-level evidence still constrain clinical decision-making. The solution lies in future in-depth research and clinical translation: on one hand, relying on novel biomarkers such as CET, the peripheral blood transcriptome, and circulating tumor DNA to achieve truly individualized dosing and risk stratification; on the other hand, actively developing more selective intervention strategies, including bispecific antibodies targeting CD47/GPC3, regulating immune metabolic checkpoints like MTHFD2, utilizing nanotechnology for targeted delivery as exemplified by AU-siRetn, and intervening in the gut–liver axis immune microenvironment.

In summary, the future management of HCC in liver transplant recipients will inevitably be a highly individualized, multidisciplinary precision medicine process. By deeply integrating a profound understanding of basic immune mechanisms with innovative clinical translation strategies, we are expected to precisely navigate the immunological crossroads, ultimately achieving the dual goals of long-term graft survival and sustained tumor control.

## Figures and Tables

**Table 1:** Recent Studies on Immune Checkpoint Inhibitor Application in Hepatocellular Carcinoma Liver Transplantation.

Author and Year	Study Design	No. of Patients	Primary Treatments	Median TLAT * (Days)	Key Findings
Tabrizian et al. [103], 2025	Prospective multicenter	43 (ITT * 117)	Nivolumab (68), atezolizumab + bevacizumab (24), pembrolizumab (21), durvalumab + tremelimumab (4); 94% combined with locoregional therapy	43	AR * rate 16.3%; 3-year ITT survival 71.1%; 3-year post-transplant survival 85%; 3-year cumulative dropout 42.6%
Tabrizian et al. [104], 2024	Prospective multicenter	17	Atezolizumab + bevacizumab (16 with locoregional therapy)	78	AR rate 11.8%; ORR 94% (CR 59%); downstaging rate 82% (within Milan criteria); 3-year survival 88.2%
Aceituno et al. [105], 2025	Retrospective multicenter	45	Nivolumab (34), atezolizumab + bevacizumab (8), other ICIs (3)	58	AR rate 17.8%; no significant difference in crude AR rate vs. non-ICI group (*p* = 0.5)
Guo et al. [106], 2024	Retrospective multicenter	83	PD-1 inhibitors (95.2%), PD-L1 inhibitors (4.8%, atezolizumab); combined with targeted/local therapy or resection	58	AR rate 27.7%; 3-year OS 84.7%
Lu et al. [107], 2024	Retrospective single center	39	Tislelizumab (11), sintilimab (10), camrelizumab (7), other ICIs (11); combined with systemic/local therapy	50	AR rate 23.1% (vs. 5% in non-ICI group); perioperative AR mortality rate: 12.8% (vs. 0% in non-ICI group)
Xu et al. [108], 2024	Retrospective multi-center	25	PD-1/PD-L1 inhibitors, PD-1/CTLA-4 bispecific (cadonilimab 4%); combined with lenvatinib + local therapy	64	AR rate 12.0%
Wang et al. [109], 2023	Retrospective multi-center	16	PD-1 inhibitors; combined with targeted/local therapy	AR group: 21; non-AR group: 60	AR rate 56.3%; no immune-related graft loss or death
Qiao et al. [110], 2021	Retrospective multi-center	7	Camrelizumab or pembrolizumab + lenvatinib	42	AR rate 14.3%; ORR 71% (mRECIST)

Abbreviations: TLAT, the time interval between the last administration of ICI therapy and LT; ITT, intention-to-treat; AR, acute rejection. The asterisk (*) is used to denote terms for which abbreviations are defined in this footnote.

**Table 2:** Clinical Trials of Innovative Strategies Aiming to Induce Operational Tolerance in Liver Transplant Recipients.

NCT Number	Phase/Enrollment	Core Intervention Strategy	Strategy Category/Key Methodology	Primary Endpoint (Safety)	Primary Endpoint (Efficacy)
NCT02260375	I/20	Single infusion of third-party bone marrow-derived mesenchymal stromal cells.	Third-party cells, non-specific immunomodulation	Number of adverse events	Exploratory biomarkers
NCT04208919	I/II/24	Infusion of donor-derived regulatory dendritic cells at 1–3 years post-transplant, followed by immunosuppression withdrawal.	Donor cells, delayed post-transplant infusion	Incidence of severe adverse events (e.g., infusion reaction, infection, rejection)	Proportion successfully weaned off immunosuppression
NCT03577431	I/II/9	Infusion of donor-derived alloantigen-specific Tregs (arTreg-CSB), combined with myeloablative conditioning and immunosuppression conversion.	Donor antigen-specific Tregs, with myeloablative conditioning	Adverse events attributed to investigational product and supportive regimen	Number of operationally tolerant participants
NCT05234190	I/II/33	Infusion of autologous, HLA-A2-targeting Chimeric Antigen Receptor T regulatory cells (QEL-001).	Autologous CAR-Tregs, engineered targeting	Dose-limiting toxicities and long-term treatment-emergent adverse events	— —
NCT07053488	I/II/90	Ex vivo CRISPR-Cas9 gene editing of donor liver (knockout of HLA class I A/B and class II via CIITA).	Donor organ gene editing, hypoimmunogenic graft	Incidence of Grade ≥ 3 treatment-emergent adverse events	Feasibility, graft failure rate
NCT06832189	Ib/20	Transition from tacrolimus to everolimus plus epoetin alfa, with concurrent immunosuppression withdrawal.	Pharmacologic intervention (mTOR inhibitor + EPO)	Proportion free of regimen-attributed opportunistic infection, malignancy, serious adverse events	— —
NCT06147375	N/A/47	Protocolized immunosuppression withdrawal in pediatric recipients to elucidate tolerance mechanisms and build a predictive model.	Pediatric population, prospective mechanistic & biomarker discovery	— —	Number of immune tolerance participants
NCT04950842	I/II/10	Infusion of induced T cells with suppressing functions (JB-101).	Induced inhibitory T cells	Safety	Operational tolerance rate

Abbreviations: CAR, Chimeric Antigen Receptor; CRISPR, Clustered Regularly Interspaced Short Palindromic Repeats; EPO, epoetin alfa; HLA, Human Leukocyte Antigen; mTOR, mechanistic target of rapamycin; Tregs, regulatory T cells.

## Abbreviations

The following abbreviations are used in this manuscript:

ACT	Adoptive Cell Therapy
APCs	Antigen-Presenting Cells
AUC	Area Under the Concentration-Time Curve
CET	Cumulative Exposure to Tacrolimus
CNI	Calcineurin Inhibitor
CTCs	Circulating Tumor Cells
CTLs	Cytotoxic T cells
DSA	Donor-Specific Antibody
EMT	Epithelial–Mesenchymal Transition
HCC	Hepatocellular Carcinoma
ICI	Immune Checkpoint Inhibitor
LSECs	Liver Sinusoidal Endothelial Cells
LT	Liver Transplantation
MDSCs	Myeloid-Derived Suppressor Cells
MMF	Mycophenolate Mofetil
OT	Operational Tolerance
SGK1	Serum/Glucocorticoid Regulated Kinase 1
TAM	Tumor-Associated Macrophage
tBPAR	treated Biopsy-Proven Acute Rejection
TCR	T Cell Receptor
TDM	Therapeutic Drug Monitoring
TME	Tumor Microenvironment
Tregs	Regulatory T Cell
TRMs	Tissue-Resident Memory T Cells
